# *G*-Subdiffusion Equation as an Anomalous Diffusion Equation Determined by the Time Evolution of the Mean Square Displacement of a Diffusing Molecule

**DOI:** 10.3390/e27080816

**Published:** 2025-07-31

**Authors:** Tadeusz Kosztołowicz, Aldona Dutkiewicz, Katarzyna D. Lewandowska

**Affiliations:** 1 Institute of Physics, Jan Kochanowski University, Uniwersytecka 7, 25-406 Kielce, Poland; 2 Department of Radiological Informatics and Statistics, Medical University of Gdańsk, Tuwima 15, 80-210 Gdańsk, Poland; 3 Faculty of Mathematics and Computer Science, Adam Mickiewicz University, Uniwersytetu Poznańskiego 4, 61-614 Poznań, Poland; szukala@amu.edu.pl; 4 Department of Physics and Biophysics, Medical University of Gdańsk, Dȩbinki 1, 80-211 Gdańsk, Poland; katarzyna.lewandowska@gumed.edu.pl

**Keywords:** g-subdiffusion, fractional Caputo derivative with respect to another function, anomalous diffusion, fractional calculus

## Abstract

Normal and anomalous diffusion processes are characterized by the time evolution of the mean square displacement of a diffusing molecule $\sigma^{2}\left(t\right)$. When $\sigma^{2}\left(t\right)$ is a power function of time, the process is described by a fractional subdiffusion, fractional superdiffusion or normal diffusion equation. However, for other forms of $\sigma^{2}\left(t\right)$, diffusion equations are often not defined. We show that to describe diffusion characterized by $\sigma^{2}\left(t\right)$, the *g*-subdiffusion equation with the fractional Caputo derivative with respect to a function *g* can be used. Choosing an appropriate function *g*, we obtain Green’s function for this equation, which generates the assumed $\sigma^{2}\left(t\right)$. A method for solving such an equation, based on the Laplace transform with respect to the function *g*, is also described.

## 1. Introduction

Anomalous diffusion models are often based on the assumption of constant parameters, which means that the structure of the medium remains constant over time. The continuous time random walk (CTRW) model has been frequently used to derive equations describing both normal and anomalous diffusion [1,2,3,4,5]. Within this model, the random walk of a single molecule is considered. The process is described by the probability density (Green’s function) $P(x,t|x_{0})$ of finding the molecule at point *x* at time *t*, with $x_{0}$ being the initial position of the molecule. In a one-dimensional unbounded homogeneous system (such a system is considered in this paper), the mean square displacement (MSD) of a molecule $\sigma^{2}$ is calculated as

(1) $$\sigma^{2}\left(t\right)=\int_{-\infty }^{\infty }{\left(x-x_{0}\right)}^{2}P\left(x,t|x_{0}\right)dx.$$

The relation

(2) $$\sigma^{2}\left(t\right)=\beta t^{\alpha }$$

is often used to define the type of diffusion. The coefficient β depends on a diffusion model.

For 0<α<1, we have subdiffusion; thus,

(3) $$\beta =\frac{2D_{\alpha }}{\Gamma (1+\alpha )},$$

where $D_{\alpha }$ is the subdiffusion coefficient measured in the units of $m^{2}/s^{\alpha }$. Subdiffusion takes place in systems where the mobility of diffusing molecules is significantly restricted, such as in gels, porous materials, and bacterial biofilms [2,6,7,8,9,10]. For α=1, we have normal diffusion with $\beta =2D_{1}$. When α>1, we have superdiffusion (facilitated diffusion); examples include diffusion in turbulent media and in random velocity fields [11,12,13,14], migration of cells [15], transport of endogeneous intracellular particles in pathogens [16], and movement of mussels [17]. However, the CTRW model gives β=∞ (see Equations (48) and (49), and the comment below them in ref. [18]), which provides $\sigma^{2}\left(t\right)=\infty$ for t>0. The superdiffusion model, based on the *g*-subdiffusion equation, which gives β<∞, is shown in refs. [18,19]. Equation (2) is complemented by the following relation, which defines slow subdiffusion (ultraslow diffusion):

(4) $$\sigma^{2}\left(t\right)∼v\left(t\right),$$

where *v* is a slowly varying function that satisfies the condition v(at)/v(t)→1 when t→∞ for any a>0. In practice, *v* typically involves combinations of logarithmic functions. Ultraslow diffusion is an extremely slow process, qualitatively distinct from ordinary subdiffusion. It has been observed, for example, in the water diffusion of aqueous sucrose glasses [20] and in language dynamics [21]. Superdiffusion is typically described by equations involving fractional derivatives with respect to space, whereas subdiffusion is modeled using time-fractional derivatives. Ultraslow diffusion is described by integro-differential equations with integral operators that are not usually identified as time-fractional derivatives [22,23,24,25,26,27,28]. The aforementioned differential equations with constant parameters are applicable to diffusion in media with time-invariant properties. Diffusion parameters depend on interactions between the diffusing molecules and their environment, as well as on the medium’s structure—both of which can evolve over time. The single molecule tracking method allows for the experimental determination of $\sigma^{2}\left(t\right)$. We mention that other power functions with respect to time have been used, which are experimentally measurable, from which subdiffusion parameters can be determined. An example of this is the time evolution of the so-called thickness of the membrane layer [8]. Processes with a time-varying diffusion exponent have been observed in the diffusion of bacteria on small beads in a freely suspended soap film [29], in the transport of colloidal particles between two parallel plates [30], microspheres in a living eukaryotic cell [31], endogenous lipid granules in living yeast cells [32], and in the diffusion of passive molecules in the active bath with moving particles [33,34]. According to the Stokes–Einstein formula, $D_{1}∼T$, the change in the temperature *T* of a liquid generates a change in the diffusion coefficient. The diffusion coefficient of chloride ions in concrete has been observed to vary over time [35,36]. Other examples include $D_{\alpha }\left(t\right)∼\log\left(t\right)$, which is caused by the aging process of a complex system in which anomalous diffusion occurs [37], and $D_{\alpha }\left(t\right)=ae^{\pm 2bt}$, where *a* and *b* are constant parameters [38]. An experimental study outlined that the diffusion model with power law $D_{\alpha }\left(t\right)$ well describes water diffusion in brain tissues [39,40]. The function $D_{\alpha }\left(t\right)$ can have a more complicated form, e.g., it may contain an oscillatory component. Such a dependency can occur, for example, in the diffusion of antibiotics in bacterial biofilms. Bacteria activate various defense mechanisms against the action of antibiotics [41,42]. One such mechanism is the thickening of the biofilm, which significantly impedes the diffusion of the antibiotic and reduces the diffusion coefficient. Slowing down diffusion of the antibiotic reduces the risk of the antibiotic having an effect on the bacteria. This process can cause a relaxation of the defense mechanisms of bacteria and increase the diffusion of the antibiotic. Bacteria, feeling a greater threat, intensify their defense mechanisms again, and so on. The subdiffusion coefficient of the antibiotic in the biofilm may then undergo periodic changes. Diffusion coefficients that oscillate with time, reflecting complex memory or frictional effects in the system, have been considered in some fractional diffusion models [43]. When the diffusion parameters are not constant, various equations are proposed to describe the diffusion process. These include subdiffusion equations with a time-fractional derivative whose order depends on time (and possibly on the spatial variable) [44,45,46,47,48,49,50,51,52,53,54,55], as well as equations involving a linear combination of time-fractional derivatives of different orders [56]. The above-mentioned changes in the diffusion parameters over time may cause the form of $\sigma^{2}\left(t\right)$ to be different than that expressed by Equations (2) and (4).

If the function $\sigma^{2}\left(t\right)$ has a complicated form, the question arises as to what equation describes the subdiffusion process and whether there are methods for solving such an equation. In further considerations, we use the *g*-subdiffusion equation with a fractional Caputo time derivative with respect to a function *g* [57,58]. The equation has been applied to describe smooth transition processes from subdiffusion to other types of diffusion [18,57], to model superdiffusion [19], and for drug transport in a system composed of closely packed gel beads placed in water [59]. The aim of this article is to show that the *g*-subdiffusion equation can describe any diffusion process defined by the time evolution of MSD that increases from zero to infinity. Our considerations demonstrate the universality of the *g*-subdiffusion equation.

This paper is organized as follows. In Section 2, we present the *g*-subdiffusion equation along with its interpretation. A method for solving this equation with the *g*-Laplace transform method is also discussed. We also show the explicit form of Green’s function and the dependence of $\sigma^{2}\left(t\right)$ on the function *g*. A modified CTRW model providing the *g*-subdiffusion equation is briefly described. In Section 3, diffusion processes generating four different functions $\sigma^{2}\left(t\right)$ are considered, two of which contain an oscillating component. These processes are described by the *g*-subdiffusion equation with respect to the function *g* determined by $\sigma^{2}$. Concluding remarks are presented in Section 4.

## 2. *G*-Subdiffusion Equation

The CTRW model provides the following ordinary subdiffusion equation:

(5) ∂αCP(x,t|x0)∂tα=Dα∂2P(x,t|x0)∂x2,

where 0<α<1; the Caputo fractional derivative is defined for 0<α<1 as

(6) dαCf(t)dtα=1Γ(1−α)∫0t(t−u)−αf(1)(u)du,

$f^{\left(1\right)}\left(u\right)=df\left(u\right)/du$. Formally, the normal diffusion equation

(7) $$\frac{\partial P(x,t|x_{0})}{\partial t}=D_{1}\frac{\partial^{2}P\left(x,t|x_{0}\right)}{\partial x^{2}}$$

can be treated as a special case of the subdiffusion equation for α=1. Equation (5) can be rewritten in an equivalent form using the fractional Riemann–Liouville time derivative of the order 1−α [2,3,4,7].

The *g*–subdiffusion equation can be interpreted as a modified version of Equation (5)), where the modification involves replacing the time variable *t* with a function g(t), t↦g(t). The function g(t) is expressed in units of time and satisfies the conditions g(0)=0, g(∞)=∞, and $g^{\left(1\right)}\left(t\right)>0$. The *g*–subdiffusion equation is [57,58]

(8) ∂gαCP(x,t|x0)∂tα=Dα∂2P(x,t|x0)∂x2,

where the *g*-Caputo fractional derivative of the order α with respect to the function *g* is defined for 0<α<1 as [60]

(9) dgαCf(t)dtα=1Γ(1−α)∫0t(g(t)−g(u))−αf(1)(u)du.

When g(t)=t, the *g*-Caputo fractional derivative takes the form of an ordinary Caputo derivative. Green’s function $P(x,t|x_{0})$ is a solution to a subdiffusion equation for the initial conditions $P\left(x,0|x_{0}\right)=\delta \left(x-x_{0}\right)$, where δ is the delta–Dirac function. For an unbounded domain, the boundary conditions are $P(\pm \infty ,t|x_{0})=0$. To solve the *g*-subdiffusion equation, we use the *g*-Laplace transform, which is defined as follows [61,62]:

(10) $$L_{g}\left[f\left(t\right)\right]\left(s\right)=\int_{0}^{\infty }e^{-sg(t)}f\left(t\right)g^{\prime }\left(t\right)dt.$$

The *g*–Laplace transform is related to the ordinary Laplace transform $L\left[f\left(t\right)\right]\left(s\right)=\int_{0}^{\infty }e^{-st}f\left(t\right)dt$,

(11) $$L_{g}\left[f\left(t\right)\right]\left(s\right)=L\left[f\left(g^{-1}\left(t\right)\right)\right]\left(s\right).$$

Equation (11) provides the relation

(12) $$L_{g}\left[f\left(t\right)\right]\left(s\right)=L\left[h\left(t\right)\right]\left(s\right)\Leftrightarrow f\left(t\right)=h\left(g\left(t\right)\right).$$

The above formula facilitates the computation of the inverse *g*–Laplace transform, provided that the inverse of the standard Laplace transform is known. For example, since $L^{-1}\left[1/s^{1+\nu }\right]\left(t\right)=t^{\nu }/\Gamma \left(1+\nu \right)$, ν>−1, and $L^{-1}\left[s^{\nu }e^{-as^{\mu }}\right]\left(t\right)=t^{-1-\nu }\sum_{k=0}^{\infty }\left(1/k!\Gamma \left(-\nu -\mu k\right)\right){\left(-a/t^{\mu }\right)}^{k}$, a,μ>0, we get [63,64]

(13) $$L_{g}^{-1}\left[\frac{1}{s^{1+\nu }}\right]\left(t\right)=\frac{g^{\nu }\left(t\right)}{\Gamma (1+\nu )}\;,\;\nu >-1,$$

and

(14) $$\begin{array}{c} L_{g}^{-1}\left[s^{\nu }e^{-as^{\mu }}\right]\left(t\right)=\frac{1}{g^{1+\nu }\left(t\right)}\sum_{k=0}^{\infty }\frac{1}{k!\Gamma (-\nu -\mu k)}{\left(-\frac{a}{g^{\mu }\left(t\right)}\right)}^{k}\equiv f_{\nu ,\mu }\left(g\left(t\right);a\right), \end{array}$$

a,μ>0. The function $f_{\nu ,\mu }$ is a special case of the Wright function and the H-Fox function. The calculations involved in solving Equation (8) using the *g*–Laplace transform are analogous to those used in solving Equation (5) by means of the ordinary Laplace transform method. Due to the relation [61,62]

(15) LgdgαCf(t)dtα(s)=sαLg[f(t)](s)−sα−1f(0),

where 0<α≤1, the *g*–Laplace transform of Equation (8) reads

(16) $$\begin{array}{c} s^{\alpha }L_{g}\left[P\left(x,t|x_{0}\right)\right]\left(s\right)-s^{\alpha -1}P\left(x,0|x_{0}\right)=D_{\alpha }\frac{\partial^{2}L_{g}\left[P\left(x,t|x_{0}\right)\right]\left(s\right)}{\partial x^{2}}. \end{array}$$

The *g*–Laplace transform of Green’s function, which is the solution to Equation (16) for the boundary conditions $L_{g}\left[P\left(\pm \infty ,t|x_{0}\right)\right]\left(s\right)=0$ that follows, is

(17) $$L_{g}\left[P\left(x,t|x_{0}\right)\right]\left(s\right)=\frac{1}{2\sqrt{D_{\alpha }}s^{1-\alpha /2}}\;e^{-\frac{|x-x_{0}|}{\sqrt{D_{\alpha }}}s^{\alpha /2}}.$$

From Equations (14) and (17), we obtain

(18) $$P\left(x,t|x_{0}\right)=\frac{1}{2\sqrt{D_{\alpha }}}f_{-1+\alpha /2,\alpha /2}\left(g\left(t\right);\frac{|x-x_{0}|}{\sqrt{D_{\alpha }}}\right).$$

The *g*–Laplace transform of Equations (1) and (17) provide $L_{g}\left[\sigma^{2}\left(t\right)\right]\left(s\right)=2D_{\alpha }/s^{1+\alpha }$. Finally, we get

(19) $$\sigma^{2}\left(t\right)=\frac{2D_{\alpha }g^{\alpha }\left(t\right)}{\Gamma (1+\alpha )}.$$

The equations describing normal and anomalous diffusion in a system with constant diffusion parameters can be derived from the CTRW model. Within this model, the diffusion of a molecule is determined by two probability densities: the waiting time for a molecule to jump ψ(Δt) and the jump length λ(Δx). For subdiffusion, ψ is a heavy-tailed distribution, which gives an infinite mean value of the time Δt, while the mean value of the jump length Δx is finite. The *g*-subdiffusion equation can be derived from the modified CTRW (ModCTRW) model, as described in detail in ref. [58]. When comparing both models, the main change in the ModCTRW model is the use of the function $\psi_{g}\left(\Delta t\right)=\psi \left(g\left(\Delta t\right)\right)$ as a function describing the waiting time for a molecule to jump. However, this function is not normalized; therefore, it is not the probability density of the variable Δt. The probability density of the waiting time for a molecule to jump for the *g*-subdiffusion process, ${\tilde{\psi }}_{g}\left(\Delta t\right)$, is given by the formula ${\tilde{\psi }}_{g}\left(\Delta t\right)=\psi \left(g^{-1}\left(\Delta t\right)\right)/g^{\left(1\right)}\left(g^{-1}\left(\Delta t\right)\right)$. However, for practical reasons, the function $\psi_{g}$ is involved in the ModCTRW model instead of ${\tilde{\psi }}_{g}$. Furthermore, ModCTRW uses the *g*-Lapcace transform $L_{g}$ and the so-called *g*-convolution of functions with respect to time, $\left(f{*}_{g}h\right)\left(t\right)=\left[\left(f\circ g^{-1}\right)*\left(h\circ g^{-1}\right)\right]\left(g\left(t\right)\right)$, instead of the ordinary Laplace transform L and the ordinary convolution of the functions $\left(f*h\right)\left(t\right)=\int_{0}^{t}f\left(t^{\prime }\right)h\left(t-t^{\prime }\right)dt^{\prime }$ used in the CTRW model, respectively. We mention that due to the relation $L_{g}\left[\left(f{*}_{g}h\right)\left(t\right)\right]\left(s\right)=L_{g}\left[f\left(t\right)\right]\left(s\right)L_{g}\left[h\left(t\right)\right]\left(s\right)$, the technique for deriving equations from the ModCTRW model is similar to that for the ordinary subdiffusion equation within the CTRW model.

## 3. Time Evolution of $\sigma^{2}$ as a Function Defining the Diffusion Process

As mentioned in Section 1, the function $\sigma^{2}\left(t\right)$ is usually used to define the type of diffusion occurring. The function is experimentally measurable. The single particle tracking method is used when a random walk of a single molecule is observed [65,66,67,68,69]. The function $\sigma^{2}\left(t\right)$ can also be determined in another way, e.g., by studying the release of a substance from one vessel to another through a thin membrane. At the initial moment, the vessel *A* contains a homogeneous solution of a diffusing substance with an initial concentration $C_{0}$, the subdiffusion parameters in the vessel are α and $D_{\alpha }$, and the vessel *B* contains a pure solvent. When the membrane allows free passage to occur from vessel *A* to *B*, and the returning of molecules though this passage is practically impossible, then the total amount of the substance *N* in the vessel *B* evolves in time as $N\left(t\right)=\eta t^{\alpha /2}$, where $\eta =C_{0}\sqrt{D_{\alpha }}/\Gamma \left(1+\alpha /2\right)$ [59]. Combining these equations with Equations (2) and (3), we get $\sigma^{2}\left(t\right)=2\Gamma^{2}\left(1+\alpha /2\right)N^{2}\left(t\right)/\left[C_{0}^{2}\Gamma \left(1+\alpha \right)\right]$. Another method is to measure the temporal evolution of the thickness of the near-membrane layer ρ(t). It is defined as the distance from the membrane to the point, where the substance concentration drops *k* times with respect to the membrane surface in the vessel *B*. We get $\sigma^{2}\left(t\right)=\eta \rho^{2}\left(t\right)$, where η is controlled by α, $D_{\alpha }$, and *k* (see [8]).

When $\sigma^{2}$ evolves in time according to Equation (2), the equations describing the diffusion process are known. However, as mentioned in Section 1, more complicated forms of $\sigma^{2}\left(t\right)$ are possible for which the equation describing the process may not be known. Based on our considerations in Section 2, we conclude that such a process can be described by the *g*-subdiffusion equation (Equation (8)), in which

(20) $$g\left(t\right)={\left(\frac{\Gamma \left(1+\alpha \right)\sigma^{2}\left(t\right)}{2D_{\alpha }}\right)}^{1/\alpha }.$$

It is also recommended for the reader to view Equation (19). Green’s function is given by Equation (18). When the molecules diffuse independently of each other, the concentration *C* of the diffusing molecules can be calculated using the following formula:

(21) $$C\left(x,t\right)=\int_{-\infty }^{\infty }P\left(x,t|x_{0}\right)C\left(x_{0},0\right)dx_{0}.$$

Then, the *g*-subdiffusion equation is also satisfied by the function C(x,t).

As an example, we consider four cases of the function $\sigma^{2}\left(t\right)$, two of which contain an oscillating component.

1. For
  (22) $$\sigma_{1}^{2}\left(t\right)=\frac{2D_{\alpha }t^{\alpha }{\left(1+at\right)}^{\kappa }}{\Gamma (1+\alpha )},$$
  we have
  (23) $$g_{1}\left(t\right)=t{\left(1+at\right)}^{\kappa /\alpha }.$$
2. When
  (24) $$\sigma_{2}^{2}\left(t\right)=\frac{2D_{\alpha }t^{\alpha }}{\Gamma \left(1+\alpha \right){\left(1+at\right)}^{\kappa }},$$
  we get
  (25) $$g_{2}\left(t\right)=\frac{t}{{\left(1+at\right)}^{\kappa /\alpha }}.$$
3. When
  (26) $$\sigma_{3}^{2}\left(t\right)=\frac{2D_{\alpha }t^{\alpha }{\left(1+at+b\sin\left(\omega t\right)\right)}^{\kappa }}{\Gamma (1+\alpha )},$$
  there is
  (27) $$g_{3}\left(t\right)=t{\left(1+at+b\sin\left(\omega t\right)\right)}^{\kappa /\alpha }.$$
4. For
  (28) $$\sigma_{4}^{2}\left(t\right)=\frac{2D_{\alpha }t^{\alpha }}{\Gamma \left(1+\alpha \right){\left(1+at+b\sin\left(\omega t\right)\right)}^{\kappa }},$$
  we get

(29) $$g_{4}\left(t\right)=\frac{t}{{\left(1+at+b\sin\left(\omega t\right)\right)}^{\kappa /\alpha }}.$$

In the above equations, it is assumed that $D_{\alpha },\kappa ,\omega >0$, α∈(0,1), and a>bω>0.

**Figure 1:**
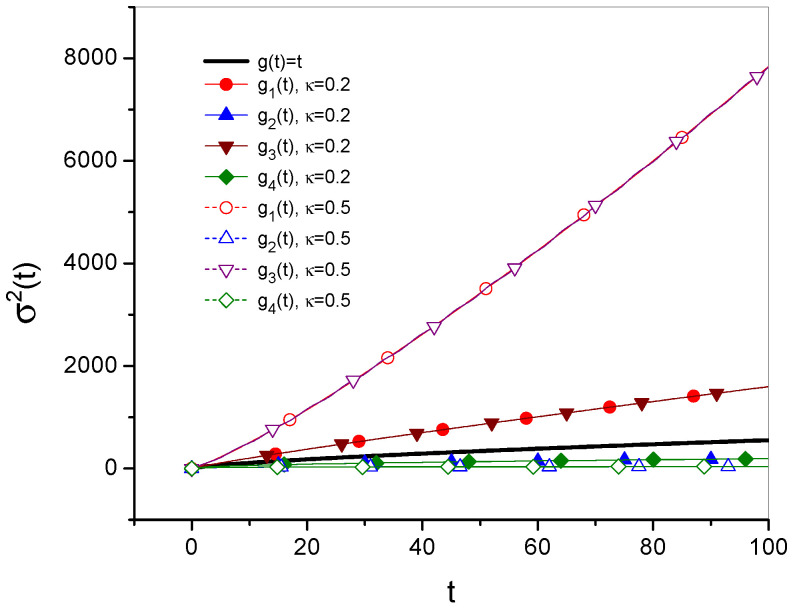
Time evolution of the MSD for the cases described in the legend. The functions $\sigma^{2}$ are determined by Equation (19) for the function $g_{i}$, where i=1,2,3,4 are given by Equations (23), (25), (27), and (29), respectively, and for κ=0.2 and κ=0.5.

**Figure 2:**
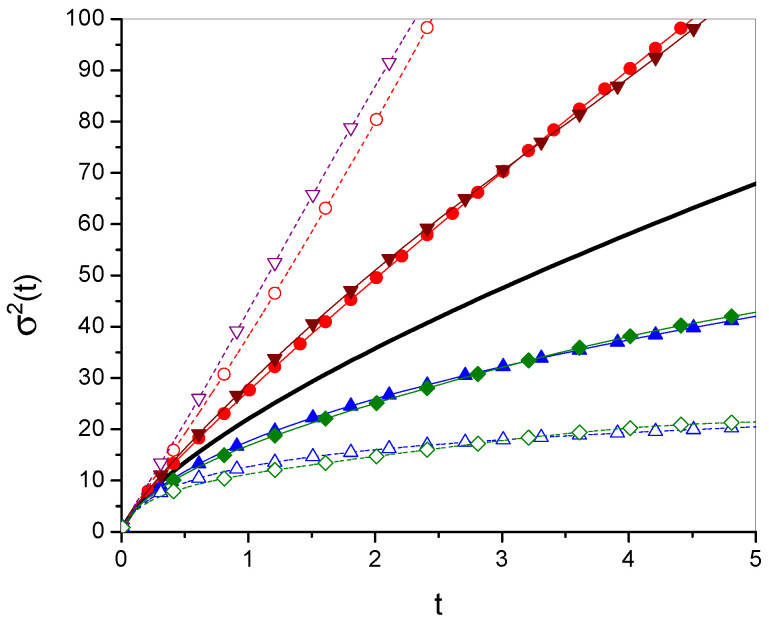
Fragment of Figure 1 depicting relatively short times. The differences in the functions generated by $g_{1}$ and $g_{3}$, as well as by $g_{2}$ and $g_{4}$, are caused by the oscillation term in $g_{3}$ and $g_{4}$; the legend, omitted in this Figure, is the same as in Figure 1.

**Figure 3:**
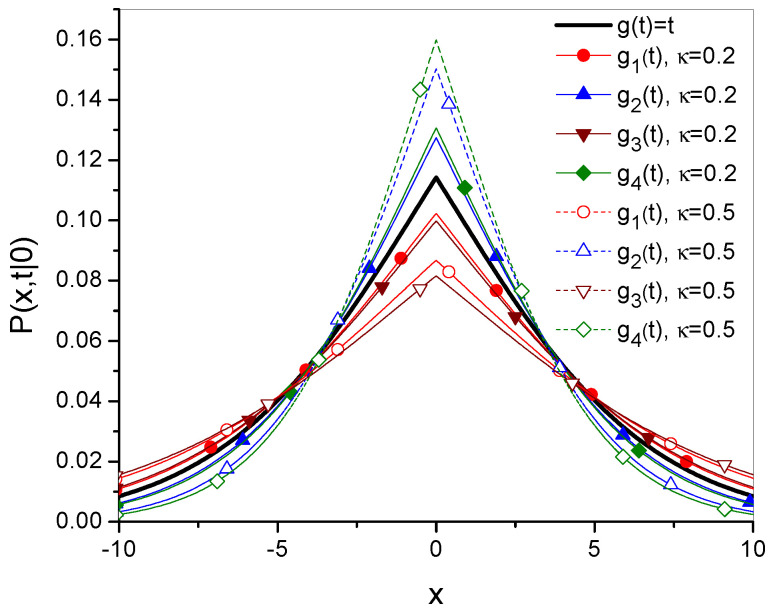
Plots of Green’s functions, showing Equation (18) for t=1, κ=0.2 and κ=0.5. The functions *P* are generated by $g_{i}$ and described in the caption of Figure 1.

**Figure 4:**
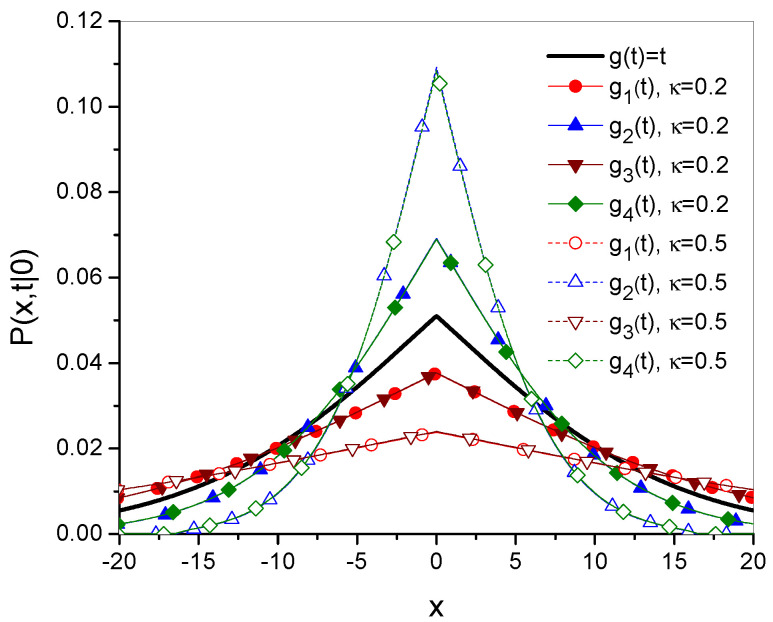
Plots of Green’s functions, showing Equation (18) for t=10; the additional description, which is omitted here, is the same as in Figure 3.

The time evolutions of the mean square displacement of diffusing particle $\sigma^{2}\left(t\right)$ are given in Figure 1 and Figure 2. Figure 3 and Figure 4 show Green’s function plots for times t=1 and t=10, respectively. The plots of the functions are compared with the functions obtained for ordinary subdiffusion with constant parameters α and $D_{\alpha }$, for which g(t)≡t and $\sigma^{2}\left(t\right)=2D_{\alpha }t^{\alpha }/\Gamma \left(1+\alpha \right)$, which are marked with a thick solid line without symbols. All plots are made for the function $g_{i}$, i=1,2,3,4, defined by Equations (23), (25), (27), and (29), respectively; the numbering of other functions is consistent with the numbering of the function $g_{i}$. The plots are made for $D_{\alpha }=10$, α=0.7, a=2, b=1, ω=1, and $x_{0}=0$; the values of all parameters are given in arbitrarily chosen units. In each case, two values of κ, namely κ=0.2 and κ=0.5, are considered. Comparing $\sigma_{1}^{2}$ and $\sigma_{2}^{2}$ with $\sigma_{3}^{2}$ and $\sigma_{4}^{2}$, respectively, in Figure 2, we see how the oscillatory effect changes $\sigma^{2}\left(t\right)$. The effect, involved in the functions $\sigma_{3}^{2}$ and $\sigma_{4}^{2}$, is visible for relatively short times. This fact can also be seen by analyzing the plots of Green’s functions. In Figure 3, for t=1, the plots of the functions generated by $\sigma_{1}^{2}$ and $\sigma_{3}^{2}$ differ from each other, and the same applies to the functions generated by $\sigma_{2}^{2}$ and $\sigma_{4}^{2}$. These differences almost disappear in Figure 4 for t=10.

## 4. Final Remarks

The main aim of this paper was to present an equation describing the diffusion process that is defined only by the time evolution of the MSD $\sigma^{2}\left(t\right)$, which increases from zero to infinity. This equation is the *g*-subdiffusion equation (Equation (8)) for the function *g* defined by Equation (20). For many processes, $\sigma^{2}\left(t\right)$ can be determined experimentally. In such a case, the diffusion equation generated by this function can be interpreted as being determined based on experimental data. The equation contains the time-fractional Caputo derivative with respect to an increasing function g(t). As g(t)≡t, the *g*-subdiffusion equation becomes the ordinary subdiffusion equation. The presented model confirms the usefulness of the *g*-subdiffusion equation in modeling anomalous diffusion processes. The parameters α and $D_{\alpha }$, involved in the equation, are determined from additional considerations. For example, the drug transport in a system containing densely packed gel beads is described by the *g*-subdiffusion equation, which is confirmed by empirical studies [59]. However, in the initial time interval, this process is well described by the ordinary subdiffusion equation (Equation (5)), for which the parameters can be determined.

The function $\sigma^{2}\left(t\right)$ is criticized as not defining the type of diffusion unambiguously. As shown in ref. [70], the appropriate combination of superdiffusion and subdiffusion effects provides the relation $\sigma^{2}\left(t\right)∼t$; however, such a process cannot be interpreted as normal diffusion. Nonetheless, $\sigma^{2}\left(t\right)$ is an important characteristic of the diffusion process. In our considerations, we use this function for defining the diffusion process, without defining the type of diffusion based on Equation (2). To sum up, the *g*-subdiffusion equation provides a useful way to describe the diffusion process when the time evolution of the MSD is different from that defined by Equations (2) and (4), especially when this function takes a complicated form. In particular, this equation seems to be useful in modeling subdiffusion processes with time-varying parameters α and/or $D_{\alpha }$.

As mentioned in Section 2, the practical use of the ModCTRW model in deriving the *g*-subdiffusion equations is similar to the derivation of the ordinary subdiffusion equations using the classical CTRW model. Following the methods of deriving subdiffusion–reaction and subdiffusion–advection equations from the latter model, one can derive more general *g*-subdiffusion–reaction and *g*-subdiffusion–advection equations. In our opinion, the use of a *g*-subdiffusion equation can be particularly effective in modeling processes previously described by ordinary subdiffusion equations with a varying parameter α, such as in the transport of antibiotics in bacterial biofilms (see [9,10] and the references cited therein) and in subdiffusion systems with rapidly oscillating boundary conditions, as in the case of subdiffusive impedance [71]. We mention that the name “*g*-subdiffusion equation” does not only refer to subdiffusion. It can describe, among others, superdiffusion understood as a process in which $\sigma^{2}\left(t\right)$ grows faster in time than a linear function.

## Data Availability

The original contributions presented in this study are included in the article. Further inquiries can be directed to the corresponding author.

## References

[1] Montroll E.W., Weiss G.H. (1965). Random walks on lattices II. J. Math. Phys..

[2] Metzler R., Klafter J. (2000). The random walk’s guide to anomalous diffusion: A fractional dynamics approach. Phys. Rep..

[3] Compte A. (1996). Stochastic foundations of fractional dynamics. Phys. Rev. E.

[4] Klafter J., Sokolov I.M. (2011). First Step in Random Walks. From Tools to Applications.

[5] Barkai E., Metzler R., Klafter J. (2000). From continuous time random walks to the fractional Fokker-Planck equation. Phys. Rev. E.

[6] Bouchaud J.P., Georgies A. (1990). Anomalous diffusion in disordered media: Statistical mechanisms, models and physical applications. Phys. Rep..

[7] Metzler R., Klafter J. (2004). The restaurant at the end of the random walk: Recent developments in the description of anomalous transport by fractional dynamics. J. Phys. A.

[8] Kosztołowicz T., Dworecki K., Mrówczyński S. (2005). How to measure subdiffusion parameters. Phys. Rev. Lett..

[9] Kosztołowicz T., Metzler R. (2020). Diffusion of antibiotics through a biofilm in the presence of diffusion and absorption barriers. Phys. Rev. E.

[10] Kosztołowicz T., Metzler R., Wa̧sik S., Arabski M. (2020). Modelling experimentally measured of ciprofloxacin antibiotic diffusion in *Pseudomonas aeruginosa* biofilm formed in artificial sputum medium. PLoS ONE.

[11] Redner S. (1989). Superdiffusive transport due to random velocity fields. Phys. D Nonlin. Phenom..

[12] Zumofen G., Klafter J., Blumen A. (1990). Enhanced diffusion in random velocity fields. Phys. Rev. A.

[13] Bouchaud J.P., Georges A., Koplik J., Provata A., Redner S. (1990). Superdiffusion in random velocity fields. Phys. Rev. Lett..

[14] Compte A., Cáceres M.O. (1998). Fractional dynamics in random velocity fields. Phys. Rev. Lett..

[15] Dieterich P., Klages R., Preuss R., Schwab A. (2008). Anomalous dynamics of cell migration. Proc. Natl. Acad. Sci. USA.

[16] Reverey J.F., Jeon J.H., Bao H., Leippe M., Metzler R., Selhuber-Unkel C. (2015). Superdiffusion dominates intracellular particle motion in the supercrowded cytoplasm of pathogenic *Acanthamoeba castellanii*. Sci. Rep..

[17] de Jager M., Weissing F.J., Herman P.M.J., Nolet B.A., van de Koppel J. (2011). Lévy walks evolve through interaction between movement and environmental complexity. Science.

[18] Kosztołowicz T. (2023). Subdiffusion equation with fractional Caputo time derivative with respect to another function in modeling transition from ordinary subdiffusion to superdiffusion. Phys. Rev. E.

[19] Kosztołowicz T. (2025). Subdiffusion equation with fractional Caputo time derivative with respect to another function in modeling superdiffusion. Entropy.

[20] Zorbist B., Soonsin V., Luo B.P., Krieger U.K., Marcolli C., Peter T., Koop T. (2011). Ultra-slow water diffusion in aqueous sucrose glasses. Phys. Chem. Chem. Phys..

[21] Watanabe H. (2018). Empirical observations of ultraslow diffusion driven by the fractional dynamics in languages. Phys. Rev. E.

[22] Chechkin A.V., Klafter J., Sokolov I.M. (2003). Fractional Fokker-Planck equation for ultraslow kinetics. Europhys. Lett..

[23] Chechkin A.V., Kantz H., Metzler R. (2017). Ageing effects in ultraslow continuous time random walks. Eur. Phys. J. B.

[24] Denisov S.I., Kantz H. (2011). Continuous-time random walk with a superheavy-tailed distribution of waiting times. Phys. Rev. E.

[25] Denisov S.I., Yuste S.B., Bystrik Y.S., Kantz H., Lindenberg K. (2011). Asymptotic solutions of decoupled continuous-time random walks with superheavy-tailed waiting time and heavy-tailed jump length distributions. Phys. Rev. E.

[26] Kosztołowicz T. (2015). Subdiffusive random walk in a membrane system. J. Stat. Mech..

[27] Sanders L.P., Lomholt M.A., Lizana L., Fogelmark K., Metzler R., Abjörnsson T. (2014). Severe slowing-down and universality of the dynamics in disordered interacting many-body systems: Ageing and ultraslow diffusion. New J. Phys..

[28] Bodrova A.S., Chechkin A.V., Cherstvy A.G., Metzler R. (2015). Ultraslow scaled Brownian motion. New J. Phys..

[29] Wu X.L., Libchaber A. (2000). Particle diffusion in a quasi-two-dimensional bacterial bath. Phys. Rev. Lett..

[30] Chakrabarty A., Konya A., Wang F., Selinger J.V., Sun K., Wei Q.H. (2013). Brownian motion of boomerang colloidal particles. Phys. Rev. Lett..

[31] Caspi A., Granek R., Elbaum M. (2002). Enhanced diffusion in active intracellular transport. Phys. Rev. Lett..

[32] Jeon J.H., Tejedor V., Burov S., Barkai E., Selhuber-Unkel C., Berg-Sørensen K., Oddershede L., Metzler R. (2011). In vivo anomalous diffusion and Weak ergodicity breaking of lipid granules. Phys. Rev. Lett..

[33] Miño G., Mallouk T.E., Darnige T., Hoyos M., Dauchet J., Dunstan J., Soto R., Wang Y., Rousselet A., Clement E. (2011). Enhanced diffusion due to active swimmers at a solid surface. Phys. Rev. Lett..

[34] Bechinger C., Di Leonardo R., Löwen H., Reichhard C., Volpe G., Volpe G. (2016). Active particles in complex and crowded environments. Rev. Mod. Phys..

[35] Ma J., Yang Q., Wang X., Peng X., Qin F. (2025). Review of Prediction Models for Chloride Ion Concentration in Concrete Structures. Buildings.

[36] Luping T., Nilsson L.O. (1992). Chloride diffusivity in high strength concrete at different ages. Nord. Concr. Res..

[37] Lomholt M.A., Lizana L., Metzler R., Ambjornsson T. (2013). Microscopic origin of the logarithmic time evolution of aging processes in complex systems. Phys. Rev. Lett..

[38] Cherstvy A.G., Safdari H., Metzler R. (2021). Anomalous diffusion, nonergodicity, and ageing for exponentially and logarithmically time-dependent diffusivity: Striking differences for massive versus massless particles. J. Phys. D Appl. Phys..

[39] Novikov D.S., Fieremans E., Jespersen S.N., Kiselev V.G. (2019). Quantifying brain microstructure with diffusion MRI: Theory and parameter estimation NMR. NMR Biomed..

[40] Lee H.-H., Papaioannou A., Kim S.-L., Novikov D.S., Fieremans E. (2020). Probing axonal swelling with time dependent diffusion MRI. Commun. Biol..

[41] Anderson G.G., O’Toole G.A. (2008). Innate and Induced Resistance Mechanisms of Bacterial Biofilms. Bacterial Biofilms.

[42] Mah T.F.C., O’Toole G.A. (2001). Mechanisms of biofilm resistance to antimicrobial agents. Trends Microbiol..

[43] Bao J.D. (2021). Time-dependent fractional diffusion and friction functions for anomalous diffusion. Front. Phys..

[44] Sun H.G., Chen W., Chen Y. (2009). Variable-order fractional differential operators in anomalous diffusion modeling. Phys. A.

[45] Sun H.G., Chen W., Sheng H., Chen Y. (2010). On mean square displacement behaviors of anomalous diffusions with variable and random orders. Phys. Lett. A.

[46] Sun H.G., Chen W., Li C., Chen Y. (2012). Finite difference schemes for variable-order time fractional diffusion equation. Int. J. Bifurcat. Chaos.

[47] Chen W., Zhang J., Zhang J. (2013). A variable-order time-fractional derivative model for chloride ions sub-diffusion in concrete structure. Fract. Calc. Appl. Anal..

[48] Sun H.G., Chang A., Zhang Y., Chen W. (2019). A review on variable-order fractional differential equations: Mathematical foundations, physical models, and its applications. Fract. Calc. Appl. Anal..

[49] Yang Z., Zheng X., Wang H. (2020). A variably distributed-order time-fractional diffusion equation: Analysis and approximation. Comput. Methods Appl. Mech. Engrg..

[50] Liang Y., Wang S., Chen W., Zhou Z., Magin R.L. (2019). A survey of models of ultraslow diffusion in heterogeneous materials. Appl. Mech. Rev..

[51] Roth P., Sokolov I.M. (2020). Inhomogeneous parametric scaling and variable-order fractional diffusion equations. Phys Rev. E.

[52] Awad E., Sandev T., Metzler R., Chechkin A. (2021). Closed-form multi-dimensional solutions and asymptotic behaviors for subdiffusive processes with crossovers: I. Retarding case. Chaos Solit. Fract..

[53] Awad E., Metzler R. (2020). Crossover dynamics from superdiffusion to subdiffusion: Models and solutions. Fract. Calc. Appl. Anal..

[54] Patnaik S., Hollkamp S., Semperlotti J.P. (2020). Applications of variable-order fractional operators: A review. Proc. R. Soc. A.

[55] Fedotov S., Han D. (2009). Asymptotic behavior of the solution of the space dependent variable order fractional diffusion equation: Ultraslow anomalous aggregation. Phys. Rev. Lett..

[56] Bazhlekova E. (2021). Completely monotone multinomial Mittag–Leffler type functions and diffusion equations with multiple time-derivative. Fract. Calc. Appl. Anal..

[57] Kosztołowicz T., Dutkiewicz A. (2021). Subdiffusion equation with Caputo fractional derivative with respect to another function. Phys. Rev. E.

[58] Kosztołowicz T., Dutkiewicz A. (2021). Stochastic interpretation of *g*-subdiffusion process. Phys. Rev. E.

[59] Kosztołowicz T., Dutkiewicz A., Lewandowska K.D., Wa̧sik S., Arabski M. (2022). Subdiffusion equation with Caputo fractional derivative with respect to another function in modelling diffusion in a complex system consisting of matrix and channels. Phys. Rev. E.

[60] Almeida R. (2017). A Caputo fractional derivative of a function with respect to another function. Commun. Nonlinear Sci. Numer. Simulat..

[61] Jarad F., Abdeljawad T. (2020). Generalized fractional derivatives and Laplace transform. Discret. Contin. Dyn. Syst. Ser. S.

[62] Jarad F., Abdeljawad T., Rashid S., Hammouch Z. (2020). More properties of the proportional fractional integrals and derivatives of a function with respect to another function. Adv. Differ. Equ..

[63] Kosztołowicz T. (2004). From the solutions of diffusion equation to the solutions of subdiffusive one. J. Phys. A Math. Gen..

[64] Fahad H.M., Rehman M., Fernandez A. (2021). On Laplace transforms with respect to functions and their applications to fractional differential equations. Math Meth. Appl Sci..

[65] Weiss M. (2019). Resampling single-particle tracking data eliminates localization errors and reveals proper diffusion anomalies. Phys. Rev. E.

[66] Michalet X., Berglund A.J. (2012). Optimal diffusion coefficient estimation in single-particle tracking. Phys. Rev. E.

[67] Kepten E., Bronshtein I., Garini Y. (2013). Improved estimation of anomalous diffusion exponents in single-particle tracking experiments. Phys. Rev. E.

[68] Bailey M.L.P., Yan H., Surovtsev I., Williams J.F., King M.C., Mochrie S.G.J. (2021). Covariance distributions in single particle tracking. Phys. Rev. E.

[69] Kettmayer C., Gratton E., Estrada L.C. (2024). Comparison of MSD analysis from single particle tracking with MSD from images. Getting the best of both worlds. Methods Appl. Fluoresc..

[70] Dybiec B., Gudowska-Nowak E. (2009). Discriminating between normal and anomalous random walks. Phys. Rev. E.

[71] Kosztołowicz T., Lewandowska K.D. (2009). Hyperbolic subdiffusive impedance. J. Phys. A Math. Theor..

